# Efficacy and Safety of Nitrous Oxide and Midazolam Sedation Protocols in Pediatric Dentistry: A Multicenter Clinical Retrospective Study

**DOI:** 10.3390/children13070956

**Published:** 2026-07-20

**Authors:** Lucia Giannini, Niccolò Cenzato, Gregorio Menozzi, Alessandra Gianfiori, Gianna Dipalma, Cinzia Maspero

**Affiliations:** 1 Fondazione IRCCS Ca’ Granda Ospedale Maggiore Policlinico, 20133 Milan, Italygregorio.menozzi@unimi.it (G.M.); alessandra.gianfiori@unimi.it (A.G.); cinzia.maspero@unimi.it (C.M.); 2 Department of Biomedical, Surgical and Dental Sciences, University of Milan, 20122 Milan, Italy; g.dipalma@unilink.it; 3 Department of Interdisciplinary Medicine, University of Bari Aldo Moro, 70121 Bari, Italy; 4 Department of Life Science, Health and Health Professions, Link Campus University, 00165 Rome, Italy

**Keywords:** conscious sedation, pediatric dentistry, nitrous oxide sedation, special needs patients, midazolam, dental anxiety

## Abstract

**Highlights:**

**What are the main findings?**

- Outpatient conscious sedation with titrated nitrous oxide/oxygen, alone or combined with midazolam, enabled the successful completion of all planned complex dental and minor oral surgical procedures in selected uncooperative pediatric and special needs patients.
- No procedure required conversion to deep intravenous sedation or general anesthesia, and no major adverse events or airway emergencies were observed.

**What are the implications of the main findings?**

- Appropriate patient selection, behavioral management and clinician expertise may allow complex pediatric dental procedures to be completed under conscious sedation in selected patients.
- Dedicated outpatient Conscious Sedation Units may provide an additional treatment pathway for selected pediatric patients; however, their effect on access to care, hospital-resource utilization, and costs requires dedicated comparative evaluation.

**Abstract:**

Aim: This two-center retrospective observational study evaluated whether outpatient conscious sedation protocols based on titrated nitrous oxide/oxygen, midazolam, or their combination enabled complex dental and minor oral surgical procedures to be completed in uncooperative pediatric and special needs patients, without escalation to deep intravenous sedation or general anesthesia. Materials and Methods: Forty pediatric and special needs patients treated at the Conscious Sedation Units of Fondazione IRCCS Ca’ Granda Ospedale Maggiore Policlinico (Milan, Italy) and Policlinico of Bari (Bari, Italy) were included. Overall, 60 complex dental or minor oral surgical procedures were performed. Procedures were classified according to the sedation protocol administered: Protocol A, nitrous oxide/oxygen inhalation sedation alone; Protocol B, midazolam alone; and Protocol C, combined midazolam and nitrous oxide/oxygen sedation. Vital signs were monitored before, during, and after treatment. The primary outcome was successful procedural completion, defined as completion of the planned procedure without premature termination, escalation to deep intravenous sedation or general anesthesia, or the need for unplanned airway or emergency medical intervention. Results: Forty patients underwent 60 procedures. The mean age was 8.7 ± 2.3 years (range, 4–14 years). Protocol A was used in 24 procedures (40%), Protocol B in 6 procedures (10%), and Protocol C in 30 procedures (50%). All procedures were completed successfully, with no conversion to general anesthesia, no airway crises, and no medical emergencies. Transient elevations in heart rate and blood pressure during local anesthesia occurred in 11/60 procedures (18.3%), and mild self-limiting nausea or fatigue occurred in 5/60 procedures (8.3%). Conclusions: The evaluated protocols were associated with successful procedural completion and few recorded adverse events in this selected cohort. Combined behavioral and pharmacological management was associated with completion of the planned procedures without conversion to deep intravenous sedation or general anesthesia. Comparative studies are required to determine whether these protocols reduce general-anesthesia utilization.

## 1. Introduction

Dental fear, anxiety, and non-cooperative behavior represent significant barriers to providing adequate oral healthcare in pediatric dentistry [1,2,3,4]. When advanced dental decay or the need for minor oral surgery arises, conventional behavior management techniques may prove insufficient, frequently leading clinicians to refer uncooperative or medically compromised children to hospital settings for general anesthesia (GA) or deep intravenous sedation [3,4,5,6]. This approach, while effective in ensuring patient immobilization, carries inherent anesthetic risks, requires specialized hospital infrastructure, and imposes a substantial psychological burden on both the child and their family [5,6,7]. This retrospective two-center study reports on pediatric and special needs patients managed at the Conscious Sedation Units of Fondazione IRCCS Ca’ Granda Ospedale Maggiore Policlinico (Milan, Italy) and Policlinico of Bari (Bari, Italy) for dental extractions, surgical removal of impacted supernumerary teeth, root remnants, and complex restorative procedures.

While mild fear and anxiety represent physiological milestones in a child’s normal psychological development, they become pathological when disproportionate to the actual threat, leading to treatment avoidance [8,9,10]. Dental Fear and Anxiety (DFA) is defined as a specific emotional reaction to perceived threatening stimuli within the dental environment, often accompanied by a sense of loss of control [8,9]. A more severe manifestation is Dental Phobia (DP), characterized by persistent, marked, and irrational anxiety triggered by clearly identifiable objects such as needles, high-speed turbines, or the dental setting as a whole [9,10,11]. If left unmanaged during childhood, these psychological barriers may persist into adulthood, establishing a vicious cycle in which dental avoidance contributes to worsening oral and dentofacial conditions, potentially increasing the need for more complex orthodontic, orthopedic, or surgical interventions; these procedures, although clinically effective, may be perceived as more invasive and demanding by anxious patients, thereby further reinforcing fear and avoidance behaviors [10,11,12]. Complex oral and dentofacial conditions in growing patients may require multidisciplinary treatment involving pediatric dentists, orthodontists, oral surgeons, and other healthcare professionals [10].

In the clinical literature, dental anxiety, fear, and phobia are frequently used interchangeably to describe strong negative emotional responses associated with dental treatment, often culminating in Dental Behavior Management Problems (DBMP) [8,9,10]. Managing these challenges requires a multidisciplinary effort, in which the clinician’s ability to establish trust is as crucial as the pharmacological intervention itself [13]. A multidisciplinary approach involving pediatric dentists, oral surgeons, orthodontists, and other specialists has been advocated to optimize treatment outcomes in growing patients with complex oral conditions [14,15,16]. Inhalation conscious sedation (ICS) using a titrated mixture of nitrous oxide and oxygen (N_2_O/O_2_) has therefore been widely implemented to support pediatric dental treatment [3,4,5,6,17]. However, inhalation sedation alone cannot be considered a universal solution for severe dental phobia. In highly phobic patients, the nasal mask and the sensory changes induced by nitrous oxide may occasionally increase feelings of claustrophobia or loss of control [17,18]. Optimal results are therefore achieved when the pharmacological approach is combined with advanced behavioral management, progressive desensitization, and non-pharmacological anxiety-control strategies [17,18,19].

Furthermore, the transition from standard outpatient care to operating room procedures represents a critical bottleneck for public healthcare systems, often resulting in prolonged waiting lists and increased expenditure; outpatient dental care encompasses a wide range of increasingly complex procedures that require careful treatment planning, multidisciplinary expertise, and optimized clinical workflows to maximize efficiency and preserve hospital resources [18,20,21].

Although the safety and anxiolytic effects of nitrous oxide and midazolam are well documented, limited real-world evidence is available regarding the ability of individualized, non-intravenous outpatient sedation protocols to prevent escalation to deep intravenous sedation or general anesthesia during complex treatment in highly uncooperative pediatric and special needs patients. A previous single-center pilot study of our research group focused primarily on changes in physiological parameters and anxiety during pediatric oral surgery performed under nitrous oxide-based basic sedation or advanced sedation involving intravenous midazolam [3]. This two-center retrospective study addresses a different clinical and organizational question: evaluating procedure completion, adverse events, cooperation, recovery, and avoidance of deep intravenous sedation or general anesthesia across a range of complex dental and minor oral surgical procedures performed using nitrous oxide, oral or intranasal midazolam, or their combination.

The primary objective of this study was to evaluate whether outpatient conscious sedation protocols using nitrous oxide alone or combined with enteral or transmucosal midazolam enabled completion of complex dental and minor oral surgical procedures without escalation to deep intravenous sedation or general anesthesia. Healthcare costs, hospital-resource utilization, and comparative effectiveness versus general anesthesia were not assessed [3,20,21,22].

Secondary outcomes included intraoperative cooperation, physiological changes, recorded adverse events, postoperative recovery, changes in situational anxiety, the need for supportive physical guidance, and procedure duration.

## 2. Materials and Methods

### 2.1. Patient Selection and Study Design

A total of 40 pediatric and special needs patients who underwent 60 separate dental and surgical procedures were included in this retrospective clinical evaluation. The mean age of the patients was 8.7 ± 2.3 years, with an age range of 4–14 years.

The inclusion criteria were age between 4 and 14 years; severe non-cooperative behavior secondary to extreme dental anxiety, dental phobia, or neurodevelopmental disorders; physical status classified as ASA I, ASA II, or controlled ASA III according to the American Society of Anesthesiologists [23]; and referral to a Conscious Sedation Unit because of previous documented treatment failures in primary care outpatient clinics.

The exclusion criteria were spontaneous cooperation, anatomical or pathological upper airway obstruction preventing effective nasal breathing, confirmed vitamin B12 or folic acid deficiency, metabolic disorders interfering with nitrous oxide pathways, acute respiratory disease, severe rhinitis, middle ear infection, severe sleep apnea, or severe neuromuscular disorders with potential compromise of respiratory drive.

Patients were retrospectively identified from the clinical records of the Conscious Sedation Units at the two participating centers. All consecutive patients who underwent dental treatment under one of the investigated sedation protocols and fulfilled the predefined eligibility criteria were screened for inclusion. Cases were not selected according to treatment outcome, cooperation, or the occurrence of adverse events. Patients were excluded when they did not meet the eligibility criteria or when the available records lacked the data required for the predefined study outcomes. The patients and procedures included in the present study did not overlap with those included in our previous publication.

### 2.2. Clinical-Operational Levels of Sedation

Grade I (minimal sedation/anxiolysis): a drug-induced state during which patients respond normally to verbal commands. Cognitive function and physical coordination may be slightly impaired, but airway reflexes, spontaneous ventilation, and cardiovascular functions remain unaffected [24]. In this study, Grade I sedation corresponded to exclusive N_2_O/O_2_ inhalation sedation titrated to the patient’s individual baseline.

Grade II (moderate conscious sedation): a deeper drug-induced depression of consciousness in which patients remain drowsy but respond purposefully to verbal commands, either alone or accompanied by light tactile stimulation. Airway patency, spontaneous ventilation, and cardiovascular functions are maintained [24]. In this protocol, Grade II sedation was achieved through midazolam, administered orally or intranasally, either alone or combined with titrated N_2_O/O_2_.

### 2.3. Preoperative Protocol and Informative Visit

Every patient underwent an initial screening and informative consultation. Depending on the child’s baseline cooperation, clinicians either performed an initial N_2_O/O_2_ titration test to identify the individual therapeutic concentration or limited the session to a psychological interview and physical examination, postponing titration to a subsequent appointment. Preoperative diagnostic imaging included digital panoramic radiography and cone-beam computed tomography only when clinically indicated for treatment planning.

Before each elective sedation session, parents or legal guardians received written instructions. Solid foods, milk and other nonclear liquids were withheld for at least 6 h before sedation, while heavy or fatty meals were withheld for at least 8 h. Clear liquids were permitted until 2 h before the procedure. Compliance with these instructions was confirmed before treatment, and the procedure was postponed if these requirements had not been met.

Comprehensive informed consent was obtained from parents or legal guardians and covered the dental or surgical procedures, the pharmacological sedation modalities, the use of clinical and radiographic data for scientific purposes while ensuring anonymity, and pre- and postoperative behavioral instructions.

The choice of the sedation protocol (N_2_O/O_2_ inhalation sedation alone, midazolam alone, or combined sedation) was made on an individual basis by the sedation-trained clinician after assessment of the patient’s age, medical and developmental status, baseline level of cooperation, previous sedation experience, and the expected complexity and duration of the planned procedure. Combined sedation was generally considered for patients in whom N_2_O/O_2_ inhalation alone was considered unlikely to provide adequate anxiolysis or behavioral control.

### 2.4. Clinical Setting and Personnel

All procedures were performed in the dedicated Conscious Sedation Units of the two participating centers. The clinical team comprised an operating pediatric dentist, a dentist trained in sedation, a dental assistant, and a nurse.

### 2.5. Management and Monitoring Toolkit

Behavioral techniques: Tell–Show–Do, progressive desensitization, positive reinforcement, voice control, distraction, non-verbal communication, and strategic parental presence or absence were used to build trust and support cooperation [25].

Pharmacological agents and devices: Midazolam dosage was calculated according to the patient’s body weight using the following formula: total dose (mg) = body weight (kg) × prescribed dose (mg/kg). For oral administration, midazolam was administered at 0.3–0.5 mg/kg, with a maximum total dose of 10 mg, approximately 15–20 min before treatment. For intranasal administration, midazolam was delivered using a mucosal atomization device at 0.2 mg/kg, with a maximum total dose of 10 mg, approximately 10–15 min before treatment.

The oral route was preferred when the child was able and willing to swallow the medication, as it was less invasive and generally better accepted. The intranasal route was selected when oral administration was refused or not feasible, particularly in children with severe noncooperation, swallowing difficulties, or neurodevelopmental conditions, or when a more rapid onset of sedation was clinically desirable. In selected cases, intranasal midazolam was administered after oral premedication when the initial clinical response was considered insufficient. In these cases, the supplementary dose was calculated according to body weight, and the cumulative midazolam dose did not exceed the predefined maximum dose. Route and dosage selection were made by the sedation-trained clinician after evaluation of the patient’s age, body weight, medical history, baseline cooperation, previous sedation experience, and expected procedural complexity.

Nitrous oxide was delivered through a calibrated relative analgesia flowmeter equipped with a fail-safe oxygen mechanism and anatomical nasal masks. Nitrous oxide administration was individually titrated according to the patient’s clinical response. Sedation was initiated at a low concentration and gradually increased until an adequate level of anxiolysis and cooperation was achieved while maintaining verbal responsiveness. The concentration was subsequently adjusted throughout the procedure according to the patient’s behavior and clinical needs. In a limited number of cases, concentrations up to 70% were required to achieve adequate anxiolysis while maintaining conscious sedation.

Local anesthesia was administered according to the clinical procedure and the individual medical characteristics of the patient. Articaine with epinephrine was generally preferred when prolonged pulpal anesthesia and local hemostasis were required, provided that no contraindication to the use of an adrenergic vasoconstrictor was identified. Mepivacaine without a vasoconstrictor was selected for shorter procedures in which prolonged anesthesia or enhanced hemostasis was not required or when the patient’s medical history, previous adverse reactions, sulfite sensitivity, cardiovascular or thyroid conditions, or concomitant pharmacological therapy suggested that epinephrine should be avoided or minimized. Medical consultation was obtained when clinically indicated. The selection was therefore individualized and based on patient-specific and procedural considerations rather than on a routine institutional protocol or a generalized risk of local tissue ischemia.

Physiological and psychological monitoring: Peripheral oxygen saturation (SpO_2_) and heart rate were monitored continuously before, during, and after treatment, while respiratory rate and non-invasive blood pressure were recorded at regular intervals. Ventilation was also assessed continuously through direct clinical observation of respiratory effort, chest excursion, airway patency, skin color, and responsiveness to verbal or light tactile stimulation. Continuous capnography and end-tidal carbon dioxide monitoring were not performed in any of the included sessions. The Venham Picture Test was administered immediately before and after treatment to quantify situational dental anxiety [26]. The Newman Postoperative Test was used after treatment to confirm recovery before discharge [27]. Behavioral response and anxiety were assessed using these standardized instruments by two sedation-trained specialists who were members of the clinical team.

This study was conducted in accordance with the Declaration of Helsinki [28] and approved by the Ethics Committee of IRCCS Istituto Oncologico Gabriella Serio (Protocol No. 1075, 16 December 2024; 1979/CEL; Study L-PRF).

### 2.6. Statistical Analysis

Patient demographic and diagnostic characteristics were summarized at the patient level. Sedation protocol, procedural completion, cooperation, physiological changes, adverse events, anxiety response, and postoperative recovery were summarized at the procedure level because these variables could differ across treatment sessions in the same patient. Some patients contributed more than one procedure; therefore, procedure-level observations were not fully independent. As the study was descriptive and no inferential comparisons or regression analyses were performed, no statistical model for within-patient clustering was applied. Procedure-level percentages should consequently be interpreted as descriptive session-level estimates. Continuous variables were summarized using means, standard deviations, and ranges, whereas categorical variables were reported as absolute frequencies and percentages. No inferential comparisons between the different sedation protocols were performed because of the descriptive design of the study and the limited sample size.

## 3. Results

### Demographic and Descriptive Data

As described in Table 1, a total of 40 patients (26 males, 14 females; mean age, 8.7 ± 2.3 years; range, 4–14 years) were successfully treated, completing 60 separate dental procedures. The study sample included patients with severe dental phobia (*n* = 18; 45%), autism spectrum disorder (*n* = 10; 25%), DiGeorge syndrome (*n* = 3; 7.5%), Fragile X syndrome (*n* = 1; 2.5%), and cognitive/intellectual disabilities (*n* = 8; 20%).

All 60 planned procedures were completed without premature conclusion, escalation to deep intravenous sedation or general anesthesia, or the need for an unplanned airway or emergency medical intervention, corresponding to a successful procedural completion rate of 100%. At the patient level, all 40 patients completed their planned treatment sessions without escalation to deep intravenous sedation or general anesthesia.

Protocol A was used in 24/60 procedures (40%), Protocol B in 6/60 procedures (10%), and Protocol C in 30/60 procedures (50%) Table 2.

Physiological parameters remained within clinically acceptable safety margins across treatment sessions. Mild, transient elevations in heart rate and blood pressure were recorded during local anesthesia in 11/60 procedures (18.3%). A temporary increase in nitrous oxide concentration up to 70% was required in 4/60 procedures (6.7%). Mild supportive physical guidance was required in 4/60 procedures (6.7%). No intraoperative or postoperative medical emergencies, airway crises, paradoxical reactions, or oxygen desaturation below 97% were recorded.

The procedures mainly consisted of tooth and retained-root extractions, including impacted supernumerary teeth, whereas restorative and endodontic procedures accounted for a smaller proportion of cases. Procedure duration ranged from 10 to 85 min (mean 25.4 min).

During active treatment, patients were calm, cooperative, and relaxed in 47/60 procedures (78.3%). Transient agitation or crying occurred in 13/60 procedures (21.7%), mainly during local anesthesia, and resolved after short clinical pauses. Postoperative recovery was uneventful in 55/60 sessions (91.7%); mild, self-limiting nausea or fatigue occurred in 5/60 sessions (8.3%). The Venham Picture Test showed a reduction in situational anxiety in 58/60 evaluable sessions (96.7%) Table 3.

Successful procedural completion was defined as completion of the planned procedure, within the planned operative session, without premature termination, escalation to deep intravenous sedation or general anesthesia, or unplanned airway or emergency medical intervention as described in Table 4, Table 5 and Table 6. 

## 4. Discussion

The present study documented a high procedural completion rate and no recorded major medical or airway emergencies in this selected cohort. By matching behavioral strategies with pharmacological protocols, all procedures in this selected cohort were completed without conversion to deep intravenous sedation or general anesthesia. However, because no general anesthesia or other control cohort was included, these findings do not demonstrate that conscious sedation reduces general-anesthesia utilization or is superior to alternative management strategies [3,29,30,31,32]. This high success rate underscores the importance of a “tailored approach,” in which the sedation protocol is dynamically adjusted to the patient’s specific emotional and cognitive profile, rather than relying on a rigid, one-size-fits-all methodology.

The present findings should be regarded as complementary to, rather than confirmatory of, our previous single-center study [3]. Whereas the earlier investigation primarily assessed pre- and post-sedation physiological parameters and anxiety during pediatric oral surgery, the present study evaluated the real-world operational ability of three non-intravenous outpatient protocols to enable complete treatment without escalation to deep intravenous sedation or general anesthesia. Its two-center design, procedure-level analysis, broader range of dental and minor surgical treatments, and inclusion of patients with different neurodevelopmental and behavioral conditions provide additional clinically relevant information. Nevertheless, because treatment allocation was individualized rather than randomized, these findings should not be interpreted as demonstrating the comparative superiority of one protocol over another.

General anesthesia guarantees immobility and eliminates intraoperative behavioral challenges, but it is associated with greater organizational complexity, the need for hospital resources, and potential cardiopulmonary risks [33,34,35]. It may also impose emotional, logistical, and economic burdens on pediatric patients and their families. In contrast, outpatient conscious sedation protocols allow rapid recovery and may permit safe discharge shortly after procedure completion when appropriate discharge criteria are met [3,30]. Furthermore, minimizing hospital admissions may reduce the risk of nosocomial infections and alleviates the psychological trauma often associated with the operating room environment for young children [34].

A clinically relevant finding of this study is that many included children had been referred to specialized sedation units after previous treatment difficulties in primary care outpatient settings. The successful completion of treatment in this cohort suggests that unsuccessful conscious sedation in primary care may reflect not only the pharmacological agent used but also case selection, operator training, behavioral management, and the clinical environment [3,29,30,31]. Nitrous oxide should not be viewed as a standalone solution; its efficacy depends on integration with communication, desensitization, positive reinforcement, and a calm, structured setting [1,3,25,30]. Therefore, specialized Conscious Sedation Units play a pivotal role as intermediate care hubs, bridging the gap between standard dental clinics and major operating theaters.

Beyond completing the immediate dental or surgical procedure, conscious sedation may help create a less traumatic treatment experience for children with severe anxiety or neurodevelopmental disorders. Reducing distress during local anesthesia, operative sounds, and surgical manipulation may support more positive future dental attitudes and improve the likelihood of subsequent cooperation [7,8,30]. By deconditioning the patient’s fear response, these protocols may contribute to the long-term goal of fostering adult patients who are compliant and receptive to preventive dental care, effectively breaking the generational cycle of dental neglect.

Complex orthodontic and dental treatments may involve oral-tissue adverse effects and therefore require careful clinical monitoring and multidisciplinary management [36]. In selected anxious or non-cooperative children, conscious sedation may facilitate completion of the required procedures while minimizing stress-related interruptions.

Technically demanding pediatric dental and orthodontic-surgical procedures, including the management of dental dilacerations [35], may require coordinated multidisciplinary care [10,11] and careful monitoring of treatment-related effects on the oral tissues [36]. In selected anxious or non-cooperative children, conscious sedation may facilitate completion of such procedures; however, these references are cited to illustrate treatment complexity and do not provide direct evidence of sedation efficacy.

The broader pediatric literature also supports the use of nitrous oxide for minor and potentially painful procedures outside the dental setting, highlighting its rapid onset, favorable recovery profile, and value in reducing procedural distress when administered by appropriately trained healthcare professionals [37,38,39,40,41,42].

From an organizational perspective, outpatient conscious sedation may provide an intermediate level of care for selected patients who cannot be managed using conventional behavioral techniques alone. However, the present study did not evaluate waiting-list duration, operating-room utilization, healthcare costs, or comparative cost-effectiveness. Therefore, any potential organizational or economic advantages should be considered hypothetical and require confirmation through dedicated health-service and cost-effectiveness studies [30,33,35]. Advanced sedation involving benzodiazepines must be performed only by clinicians with appropriate postgraduate training, monitoring equipment, emergency preparedness, and compliance with national regulations [1,2,24].

The favorable outcomes observed in the present study should be interpreted in the context of the highly specialized clinical environments in which treatment was delivered. They should not be extrapolated automatically to general dental practices, particularly where sedation-trained personnel, continuous physiological monitoring, emergency equipment, or established rescue protocols are unavailable.

### Limitations

This study has several limitations. Its retrospective design, absence of a control group, and procedure-level denominators limit causal interpretation. Additionally, the data reflect outcomes from two high-volume academic centers, which may limit the generalizability of the findings to smaller private settings. Continuous capnography was not routinely available in the participating centers. Therefore, although no oxygen desaturation, airway crisis, or clinically evident respiratory complication was recorded, brief episodes of subclinical hypoventilation cannot be completely excluded.

The retrospective identification of patients introduces a potential risk of selection and information bias. Although all consecutive eligible cases were included irrespective of treatment outcome, the sample was derived from specialized academic sedation units and consisted largely of patients referred after previous treatment difficulties. Consequently, children with more severe behavioral, neurodevelopmental, or procedural complexity may be overrepresented, limiting the generalizability of the findings. Moreover, sedation protocol allocation was based on individualized clinical judgment rather than randomization, and comparisons between protocols should therefore be interpreted cautiously. Because 40 patients contributed 60 procedures, some observations were clustered within the same patient. No statistical adjustment for within-patient correlation was performed, and patients undergoing multiple procedures contributed more weight to the procedure-level descriptive percentages. These results should therefore be interpreted as session-level outcomes and not as independent patient-level estimates.

The absence of a comparison group treated under general anesthesia, alternative sedation protocols, or conventional behavioral management prevents conclusions regarding comparative effectiveness or a reduction in general-anesthesia utilization. The finding that no included procedure required conversion to general anesthesia should therefore be interpreted as a descriptive outcome of this selected cohort rather than evidence of a causal reduction in the need for general anesthesia.

Both participating centers were highly specialized academic Conscious Sedation Units with experienced sedation-trained clinicians, multidisciplinary personnel, dedicated monitoring equipment, and established emergency protocols. These organizational and professional characteristics may have contributed substantially to the high procedural completion rate and low incidence of complications. Consequently, the findings may not be reproducible in smaller dental clinics or settings lacking comparable expertise, staffing, monitoring facilities, and rescue capabilities. The external validity of the study is therefore limited, and multicenter prospective investigations involving different levels of dental care are required. Healthcare costs, operating-room utilization, waiting-list duration, and comparative resource use were not assessed. Consequently, the present findings do not demonstrate that conscious sedation is cost-saving or that it can replace general anesthesia.

Future prospective studies should compare inhalation sedation, combined sedation, and general anesthesia using standardized measures of anxiety, cooperation, adverse events, treatment completion, parent satisfaction, and cost-effectiveness.

## 5. Conclusions

Titrated nitrous oxide/oxygen, alone or combined with midazolam in selected cases, was associated with successful procedural completion and few recorded adverse events in the specialized units included in this study. When integrated with behavioral management and delivered by appropriately trained clinicians, these protocols were associated with successful completion of the planned procedures without the need for escalation to general anesthesia in this selected cohort. Whether they reduce general-anesthesia utilization or healthcare costs cannot be determined from the present study. Controlled, prospective, multicenter studies are needed to determine whether this approach can reduce general-anesthesia utilization in broader clinical populations and to establish standardized clinical guidelines.

## Figures and Tables

**Table 1:** Demographic and clinical characteristics of the study population.

Variable	N	%
Total patients	40	100%
Male sex	26	65%
Female sex	14	35%
Mean age, years	8.7 ± 2.3	—
Age range, years	4–14	—
Severe dental phobia	18	45%
Autism Spectrum Disorder	10	25%
DiGeorge syndrome	3	7.5%
Fragile X syndrome	1	2.5%
Cognitive/intellectual disability	8	20%

**Table 2:** Distribution of sedation protocols by procedure.

Protocol	Description	Procedures, n	% of Procedures
Protocol A	Titrated N_2_O/O_2_ inhalation sedation alone	24	40%
Protocol B	Midazolam alone, oral/transmucosal	6	10%
Protocol C	Midazolam + titrated N_2_O/O_2_	30	50%
Total	All procedures	60	100%

**Table 3:** Midazolam administration route within the combined sedation protocol.

Route	N	Percentage
Oral midazolam	15/30 Protocol C procedures	50% of Protocol C; 25% of all procedures
Intranasal midazolam atomization	5/30 Protocol C procedures	16.7% of Protocol C; 8.3% of all procedures
Sequential oral + intranasal midazolam	10/30 Protocol C procedures	33.3% of Protocol C; 16.7% of all procedures
Total Protocol C	30/30 Protocol C procedures	100%

**Table 4:** Procedure-level procedural completion and safety outcomes.

Outcome	n/N	%
Successful procedural completion	60/60 procedures	100%
Conversion to deep IV sedation or general anesthesia	0/60 procedures	0%
Medical emergencies, airway crises, paradoxical reactions	0/60 procedures	0%
Transient HR/BP elevations during local anesthesia	11/60 procedures	18.3%
Temporary N_2_O increase up to 70%	4/60 procedures	6.7%
Supportive physical guidance	4/60 procedures	6.7%
SpO_2_ below 97%	0/60 procedures	0%

**Table 5:** Cooperation, psychometric response, and postoperative recovery.

Outcome	n/N	%
Calm/cooperative/relaxed during active treatment	47/60 procedures	78.3%
Transient agitation or crying, limited to the local anesthesia phase	13/60 procedures	21.7%
Side-effect-free recovery	55/60 procedures	91.7%
Mild self-limiting nausea/fatigue	5/60 procedures	8.3%
Venham anxiety reduction	58/60 evaluable sessions	96.7%
No Venham anxiety reduction	2/60 evaluable sessions	3.3%

**Table 6:** Operational characteristics.

Variable	Value
Total procedures	60
Main procedure types	Tooth/root extractions (including impacted supernumerary teeth), restorative procedures, and endodontic treatments
Procedures per patient	1.5
Procedure duration, mean	25.4 min
Procedure duration, range	10–85 min
Lowest SpO_2_ recorded under N_2_O/O_2_	≥97%

## Data Availability

The data presented in this study are available on request from the corresponding author due to patient privacy regulations and institutional data protection policies.
